# Shifting levels of peripheral inflammatory profiles as an indicator for comorbid multiple autoimmune diseases and bipolar disorder: a case report

**DOI:** 10.1186/s12888-023-04820-x

**Published:** 2023-05-29

**Authors:** Yuting Shen, Lingzhuo Kong, Jianbo Lai, Shaohua Hu

**Affiliations:** 1 Department of Psychiatry, the First Affiliated Hospital, Zhejiang University School of Medicine, Hangzhou, 310003 China; 2 The Key Laboratory of Mental Disorder’s Management in Zhejiang Province, Hangzhou, 310003 China; 3 Brain Research Institute of Zhejiang University, Hangzhou, 310003 China; 4 Zhejiang Engineering Center for Mathematical Mental Health, Hangzhou, 310003 China; 5 Department of Neurobiology, NHC and CAMS Key Laboratory of Medical Neurobiology, School of Brain Science and Brian Medicine, and MOE Frontier Science Center for Brain Science and Brain-Machine Integration, Zhejiang University School of Medicine, Hangzhou, 310003 China

**Keywords:** Autoimmune diseases, Bipolar disorder, Immunological disturbance, Drug efficacy, Case report

## Abstract

Autoimmune diseases (AID) cause inflammatory changes in the peripheral blood, which might be a predisposing factor for the development of comorbid bipolar disorder (BD). The levels of peripheral inflammatory indicators and cytokines may also serve as potential biomarkers for predicting BD susceptibility and the efficacy of antipsychotics in patients with AID. Herein, we present the case of a 43-year-old female who has suffered from AID for over 16 years and was recently diagnosed with “bipolar and related disorder due to another medical condition”.

## Background

Autoimmune diseases (AID) are characterized predominantly by disturbed peripheral cytokine and acute-phase proteins [1], which have also been detected in patients with bipolar disorder (BD) [2, 3]. Inflammation activation may be associated with acute mood episodes of BD [4]. The shared serological immunological changes of AID and BD might be a potential indicator for the development of BD, which needs to be further verified by well-designed studies. Moreover, the dynamic levels of these molecules also help to evaluate the efficacy of pharmacotherapy [4].

We report herein the varied levels of peripheral inflammatory profiles and cytokines in a woman with a history of multiple AID for over 16 years, who was diagnosed with “bipolar and related disorder due to another medical condition”, in the last three months before admission. This case revealed that immune disturbance might be a driving factor for the subsequent development of BD, and emphasized the importance of monitoring peripheral inflammatory markers in susceptible individuals.

## Case presentation

A 43-year-old female developed Behcet’s disease at the age of 28, with erythema nodosum, aphthous lesions in the mouth, painful ulcers in the genitalia, and recurrent iridocyclitis. She has suffered from a sustained feeling of drowsiness, palpitations, and paroxysmal electric shock in her extremities. The immunologic test was positive for *HLA-B51*, a genetic solid susceptibility factor for Behcet’s disease, which was found in approximately 50% of the patients [5]. Autoantibodies against endothelium and DNA were absent. She also reported a history of combined Sjögren’s syndrome, asthma, and type II diabetes, but relevant examination results were absent. She was treated with tofacitinib after being diagnosed with AID. However, her treatment compliance was poor, and the clinical symptoms were not well controlled.

In October 2022, she visited the Psychiatric Outpatient Clinic with a chief complaint of emotional instability for three months. She reported low mood, loss of pleasure and lack of motivation. While taking medical history, she also reported another two similar experiences of emotional instability, one in 2018, and the other in 2019, during which her condition of AID was also poorly controlled. In October 2018, her Behcet’s disease worsened, and she developed general pain, discharge of pus from the anus and navel, and severe external pustules, which needed to be treated with methylprednisolone. In April 2019, she developed pain in the hip and low back, pustules in the vulva, irregular menstruation, and numbness in the left lower limb, which further resulted in sleep difficulty. She had been treated with methylprednisolone and cyclosporin since early 2020, but cyclosporin was discontinued months later due to undesirable side effects. She also reported that she had been on immunosuppressive therapy intermittently in the last 2 years. Although her AID-related symptoms improved, she often stopped medication on her own because of drug intolerance. It was unclear about the family history of AID and mental illnesses since she was adopted at an early age.

**Fig. 1:**
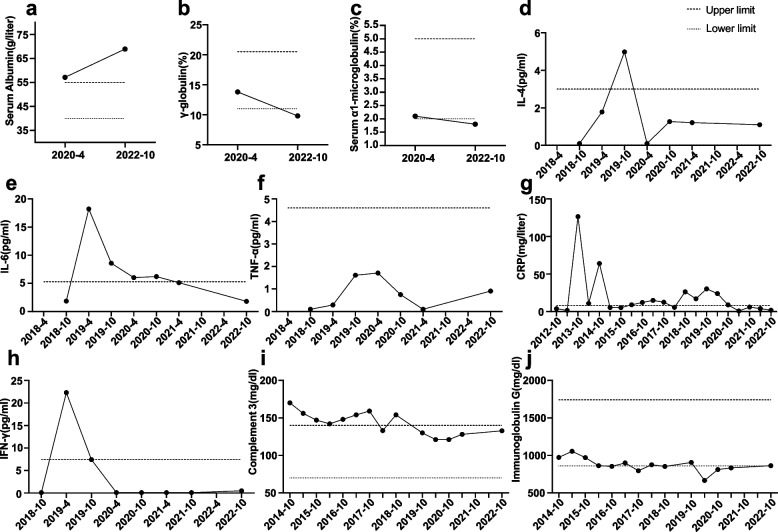
Shifting peripheral levels of inflammatory indicators and cytokines. Figure 1 shows the changing tendency of serum inflammatory indicators and cytokines of the patient. The drastic fluctuation was noted before, especially in the levels of CRP, IL-6, and complement C3. After receiving antipsychotic and immunosuppressive therapy, the serum immunological indicators returned to normal alongside the improvement in the clinical manifestations. TNF-α: tumor necrosis factor α; IFN-γ: interferon γ; IL: interleukin; CRP: C-reaction protein

After admission, physical examinations revealed Cushing’s signs, moderate vulvar ulcer, and sinus tachycardia (115 beats per minute). No apparent abnormality correlated with AID was found in the cranial magnetic resonance imaging (MRI), electroencephalogram, or serum autoantibody profiles. She refused to have her cerebrospinal fluid tested. The hematological examinations showed abnormalities in serum albumin, γ-globulin, and α1 microglobulin (Fig. 1a-c), as well as the increased level of tumor necrosis factor α (TNF-α). In addition, the results of previous examinations of circulating levels of inflammatory cytokines, C-reaction protein (CRP), and serum complement had also been well recorded (Fig. 1d-j), corresponding to her fluctuating AID conditions. In 2018 and 2019, the serum levels of CRP, interleukins (IL), TNF-α, and complement C3 varied significantly. After aforementioned immunosuppressive therapy, the levels of these inflammatory returned to normal. On psychiatric examination, the patient reported slight memory loss, exhaustion, burnout, and recurring negative perceptions. Psychometric evaluation indicated severe depression. Prior to this admission, she had been treated with venlafaxine for at least two months. However, her emotional symptoms became labile and she experienced a hypomanic episode for over ten days in the last three months, manifesting as an abnormal high mood, quick thinking, and decreased sleep needs. She also reported insomnia and auditory hallucinations.

This patient was diagnosed with “bipolar and related disorder due to another medical condition”, a depressive episode, according to the *Diagnostic and Statistical Manual of Mental Disorders, Fifth Edition*. After being hospitalized, she was treated with alprazolam 0.4 mg per night and quetiapine, which was gradually titrated up to 400 mg per night. However, her mood did not improve, and she reported difficulty in face recognition and an intolerable illusionary feeling of worm crawling. The pharmacotherapy strategy was adjusted to lurasidone 60 mg per day, quetiapine 400 mg per night, and lithium carbonate 600 mg per day. For her AID conditions, methylprednisolone 8 mg per day and cyclosporin 200 mg per day were prescribed. After 2 weeks of treatment, the clinical symptoms of both AID and BD were significantly improved, and the serum immunological profiles returned to normal (Table 1). Another 1 week later, the patient was discharged with quetiapine 300 mg per night and lurasidone 60 mg per day for maintenance therapy of BD, and methylprednisolone 8 mg per day and cyclosporin 200 mg per day for AID. Given the BD condition might link to the immune dysfunction caused by AID, quetiapine and lurasidone were gradually withdrawn 2 weeks after her discharging from hospital, and her emotion kept stable in the next half year follow-ups.

**Table 1:** Results of the patient’s latest serological examination before discharging from the hospital

Detection index	Results (reference value)
Albumin	57.5 (56–66) g/L
γ-globulin	10.1 (10–21) %
α1 microglobulin	2.1 (2–5) %
IL-4	1.10 (0–3) pg/ml
IL-6	1.80 (0–3) pg/ml
TNF-α	0.91 (0–23) pg/ml
IFN-γ	0.50 (0–18) pg/ml
CRP	1.69 (0–8) mg/L
Complement C3	123.5 (70–140) mg/dl
Immunoglobulin G	875.0 (850–1700) mg/dl

*TNF-α* Tumor necrosis factor α, *IFN-γ* Interferon γ, *IL* Interleukin, CRP C-reaction protein

This case study has been approved by the Institutional Ethical Board of the First Affiliated Hospital, Zhejiang University School of Medicine. An informed consent statement was obtained for this study.

## Discussion and conclusions

In this case study, we reported a woman with recurrent AID for 16 years and comorbid BD. The serum inflammatory factors of the patient showed significant abnormalities and noticeable fluctuation, especially when the condition of AID worsened. After being hospitalized and treated with antipsychotics and immunosuppressive medications, the serological inflammatory indicators returned to normal, as well as the clinical symptoms.

Two doubts are noteworthy in our case. First, can the emotional changes occurred before and after hospitalization be caused by AID-induced encephalitis or drug intervention? Given the evidence that 1) she released no clinical manifestations of intracranial inflammation such as epilepsy, delirium, and nuchal rigidity; 2) no encephalitis-related abnormality was observed in the MRI and electroencephalogram; 3) the serum autoantibody profiles were negative; 4) the mood episodes did not progress within the last three months. Although we did not perform the cerebrospinal fluid autoantibody profiles, the diagnosis of encephalitis was not supported. In addition, the neurotoxic manifestations of glucocorticoids and cyclosporin are mostly neural abnormal discharge (seizure) and neurodegeneration (memory deficit) [6, 7], in the context of overdose. Therefore, emotional symptoms due to immunosuppressant use was unlikely in this case. Further investigation is needed to explain the auditory hallucination and provide deeper insights into the AID-related neuropathology. Second, the diagnosis should be carefully discussed. This patient was diagnosed as “bipolar and related disorder due to another medical condition” but not “primary BD”, as 1) she showed typical depressive and manic manifestations that cannot be fully explained organically; 2) AID-induced systematic inflammation dysregulation might be crucial for BD development; 3) the manic episode might be related to the use of anti-depressant; 4) the psychopathology including insomnia and auditory hallucination is not typical for mania or hypomania. Since the BD condition was considered to be related to AID, quetiapine and lurasidone were gradually withdrawn after her mood stablized, and immunosuppressants were kept for maintenance. In summary, this report indicates that abnormalities in the immune system might predispose the development of BD in patients with AID, and monitoring the levels of peripheral immunological profiles helps evaluate the effects of BD therapy.

BD poses a severe burden to the public health system, and causes dysfunction in attention, memory, and executive functions, in a majority of patients [8]. Neuroinflammation has been recognized as one of the crucial pathogenetic mechanisms of BD [9]. AID, such as Behcet's disease, Sjögren’s syndrome, asthma, etc., has been considered to correlate with several psychiatric disorders including BD [10]. Of note, a population-based cohort study revealed a higher incidence of subsequent BD among patients with AID, and some AID diseases such as asthma have been proved as potential risk factors for the occurrence of BD [11].

Evidence has shown elevated levels of cytokines, such as TNF-α, interferon γ (IFN-γ), IL [12], complement cascade components [13], immunoglobulin [14], and inflammatory indicators such as CRP [15, 16], in BD patients compared with healthy controls. Moreover, patients with unipolar mania showed higher serum levels of IL-6 and CRP than other patients with a BD diagnosis [17]. In this case, our patient showed increased levels of CRP, complement C3 and C4, and proinflammatory cytokines such as IFN-γ, IL-4, and IL-6. Decreased levels were observed in immunoglobulin G and TNF-α (Fig. 1). In addition, it has been reported that the clinical improvement of BD correlates with the improvement of CRP [15], while the serum levels of TNF-α and other inflammatory indicators may link to the genetic susceptibility of BD [18] and poor long-term prognosis of BD including suicide [19].

In this case, the AID condition was poorly controlled before 2020, and levels of peripheral inflammatory indicators and cytokines were mostly abnormal. She had been treated with immunosuppressants since 2020, which was parallel with the improvement of AID symptoms and changes in the various serum inflammatory markers. Levels of IL-6, CRP, and immunoglobulin G were still over-expressed after 2020, probably due to her poor compliance to medications (Fig. 1). After October 2022, the markers all returned to normal except albumin, which were also detected normal in the latest serological examination (Table 1). Meanwhile, her AID symptoms further improved, and her emotions also got controlled.

Of all the indicators examined in this case, serum levels of TNF-α tended to be more closely related to Behcet’s disease [20], with IL-4 related to asthma [21] and IL-6 related to Sjögren’s syndrome [22], while TNF-α was also considered to be closely related to BD [23]. Unfortunately, we did not test for other biomarkers including IL-1, soluble IL-2 receptor, and soluble TNF receptor type 1, which are of higher specificity for AID and BD. In recent years, new therapeutic agents targeting the immune system including non-steroidal anti-inflammatory drugs [24, 25], N-acetylcysteine [26] GSK3 inhibitors [27, 28], and glucocorticoids [29, 30] have been trialed for BD treatment in pre- or clinical studies. In this case study, although methylprednisolone was used to control AID, its anti-inflammation effects might also play additional role in stabilizing the mood. Except for the anti-inflammatory medications, the two drugs for treating BD, quetiapine and lurasidone, have also been proven to suppress central [31, 32] and peripheral [33, 34] inflammatory processes. Intriguingly, non-psychotropic drugs that can change inflammatory indicators in peripheral blood have also been recently proven to regulate mental states. For example, infliximab is capable of inducing manic episodes in subjects without a preexisting BD diagnosis by downregulating the serum levels of TNF-α [35]. Meanwhile, patients with bipolar depression treated with celecoxib, a cyclooxygenase-2 inhibitor, showed decreased IL-6 and CRP levels when the clinical symptoms were improved [16]. These findings together indicated that the immune indicators and inflammatory cytokines could act as biomarkers for BD diagnosis and efficacy prediction. In addition, the combination of antipsychotic and immunosuppressive therapy proved to be effective in some autoimmune diseases, such as systemic lupus erythematosus [36], Behcet’s disease [37], autoimmune encephalomyelitis [38], and Sjögren’s syndrome [39], which in turn indicated the shared immune mechanisms between AIDs and mood disorders.

This case report provides new insights into the overlapping inflammatory disturbance in patients with comorbid AID and BD, which coordinates with the waves of symptoms and can serve as an indicator of disease relapse and remission.

## Data Availability

All data generated or analyzed during this study are included in this article.

## Abbreviations

BD: Bipolar disorder
AID: Autoimmune diseases
MRI: Magnetic resonance imaging
TNF-α: Tumor necrosis factor α
IFN-γ: Interferon γ
IL: Interleukin
CRP: C-reaction protein
